# Prevalence of human papilloma virus infection and risk of cervical intraepithelial neoplasia among female sex workers in Mumbai, India

**DOI:** 10.3332/ecancer.2024.1772

**Published:** 2024-09-19

**Authors:** Vandita Pahwa, Sharmila A Pimple, Gauravi A Mishra, Parishi Majmudar, Sanjay K Biswas, Kedar Deodhar

**Affiliations:** 1 Department of Preventive Oncology, Homi Bhabha Cancer Hospital & Research Center, New Chandigarh, Punjab, India; 2 Department of Preventive Oncology, Centre for Cancer Epidemiology (CCE), Tata Memorial Centre, Homi Bhabha National Institute (HBNI), Mumbai, India; 3 Department of Microbiology, Tata Memorial Hospital, Tata Memorial Centre, Homi Bhabha National Institute (HBNI), Mumbai, India; 4 Department of Pathology, Tata Memorial Hospital, Tata Memorial Centre, Homi Bhabha National Institute (HBNI), Mumbai, India; a https://orcid.org/0000-0002-3492-4418; b https://orcid.org/0000-0003-2976-9107; c https://orcid.org/0000-0002-0154-7302; d https://orcid.org/0000-0002-9802-0848; e https://orcid.org/0000-0002-6338-8191

**Keywords:** female sex workers, human papilloma virus (HPV), cervical cancer, CIN, screening, precancer

## Abstract

**Introduction:**

Cervical cancer, mostly caused by human papilloma virus (HPV), has disproportionately high incidence in developing countries. HPV infection being essentially a sexually transmitted infection, high-risk behaviour women with multiple sexual contacts like female sex workers (FSWs) are at higher risk of co-infection with HPV and of developing cervical precancer and cancer.

**Objective:**

This study aimed to determine the prevalence and determinants of HPV infection and cervical intraepithelial neoplasia (CIN) among FSWs in Mumbai, India.

**Methods:**

A cross-sectional study was conducted among 448 FSWs, between the ages of 18–50 years, by collaborating with local non-government organizations working for the health and welfare of FSW communities at sexually transmitted diseases clinics. All FSWs were screened for HPV DNA by hybrid capture II followed by reference diagnosis of colposcopy and/or cervical biopsy.

**Results:**

The prevalence of HPV DNA positivity was 35.5% and CIN was 2.2%. Factors significantly associated with HPV DNA positivity were age group younger than 30 years odds ratio (OR = 2.098, 95% confidence interval (CI) 1.408–3.127), Illiteracy (OR = 2.015, 95% CI 1.305–3.112), being single (OR = 2.409, 95% CI 1.558–3.724), less than 18 years of age at time of initiating work as FSW (OR = 3.718, 95% CI 3.718–2.392), having more than five clients per day (OR = 2.078, 95% CI 1.301–3.318), been working as a FSW for more than 5 years (OR = 2.321, 95% CI 1.455–3.701), not using barrier contraception methods (OR = 5.155, 95% CI 3.395–7.827) and having no exposure to human immunodeficiency virus (HIV)/acquired immunodeficiency syndrome (AIDS) education program (OR = 29.153, 95% CI 15.385–55.240). FSWs with a positive HPV DNA test are substantially more likely to have CIN compared to those with a negative test (OR = 7.6, 95% CI 1.59–36.25).

**Conclusion:**

The prevalence of HPV infection and CIN was high among FSWs. FSWs with a positive HPV DNA test had a seven times higher risk of developing CIN. The persistence of HPV infection is expected to significantly raise the risk of cervical cancer in the future. It is suggested to have an integrated approach towards cervical cancer screening and HIV/AIDS control activities.

## Introduction

Globally, cancer of the cervix ranks fourth among the most common cancers in females [1]. In addition, it has been observed that cervical cancer is disproportionally high in developing countries [2]. In India, incidence of cervical cancer is next only to breast cancer [3]. It is preventable due to vaccination and screening programs, it is curable if diagnosed early and treated in time. The most important causative agent for cervical cancer is infection with oncogenic human papilloma virus (HPV) [4, 5].

HPV is the most common sexually transmitted infection (STI). Out of the several known types of HPV, 14 are known to be carcinogenic [6]. Around two-third of carcinoma of the cervix are caused by HPV subtypes 16 and 18 [7]. Most HPV infections are transient without any symptom and resolve on their own [8]. Cervical cytological abnormalities and cancer is known to occur only with persistent HPV infection [9]. With advances in detection of HPV DNA technologies including genotyping, we can identify subgroups of population with increased risk of infection [10].

India has a dual burden of human immunodeficiency virus (HIV) infection and high cervical cancer rates. Several studies have shown that HIV positive women are at an increased risk of developing cervical squamous intraepithelial lesions (SIL) and cervical cancer [11–13]. The usual course of HPV infection is modified among HIV women. The lesions revert to normal in lesser percentage of HIV positive individuals and often expedites to severe and cancerous forms [14].

Women with high-risk behaviour (multiple sexual contacts) are at an increased risk of co-infection by HPV and intraepithelial neoplasia of the cervix. Studies reveal that HPV prevalence depends largely on age and on sexual practices [15, 16].

Studies show that female sex workers (FSWs) are known to have a very high prevalence of HPV infection primarily due to early inception age of sexual activity and prevalence of multiple sexual partners [17]. Compared to low-risk population groups, FSWs have higher vulnerability to HPV infection, thus resulting in abnormal pap smears and cervical cancer [18–20]. There is also widespread ignorance about infection spread dynamics, preventive measures and screening for cancer of the cervix [21].

The objectives of this work are to describe the determinants of, and report prevalence of high-risk HPV infection and subsequent risk of cervical intraepithelial neoplasia (CIN) among FSWs in Mumbai, India.

An increasing proportion of the FSWs in Mumbai originate from various parts of the country as well as neighbouring countries. This also introduces differences in their sociodemographic characteristics, sexual plus health-seeking behaviour and HPV prevalence and types. The knowledge of these characteristics is essential to design appropriate preventive and curative strategies for women with high-risk behaviour patterns.

## Methods

This cross-sectional study was conducted among 448 FSWs, recruited by collaborating with local non-government organizations (NGO’s) working for the health and welfare of the FSW communities at sexually transmitted diseases (STDs) clinics in Mumbai. All FSWs aged between 18 and 50 years were invited to participate in the study. Apparently, healthy FSW, non-pregnant with an intact uterus and no history of cervical cancer or debilitating physical and mental illness were recruited in the study. All FSWs who were pregnant, with a record of cervical cancer or hysterectomy, or with a debilitating condition that prevents a pelvic examination were excluded from the study.

Anticipating an HPV prevalence of 30%–50% from studies in different geographical areas, the accrual of 448 women was required in the study to assess the true prevalence of HPV among FSW at a 95% confidence interval (CI) with 80% statistical power. The study was reviewed and approved by the scientific and ethical committee of the Institutional Review Board (IRB) of Tata Memorial Hospital, Mumbai, India, and was conducted in compliance with the medical research regulations involving human subjects set by the IRB.

In the first phase of the study, the NGOs working for the health and welfare of the FSW community were identified. The study participants were recruited from community based programmes conducted by these NGO’s and attended by the FSWs. Cervical cancer awareness sessions highlighting the risk of cervical cancer due to high-risk behaviour were conducted in these programs by the medical social worker (MSW), after which the women were invited to participate in the screening programme. The FSWs were explained about the study by giving a participant information sheet. A written informed consent in the vernacular language (Hindi/Marathi) was obtained from the participant and a unique participant identification number was assigned to the eligible women.

### Design of the questionnaire

The questionnaire was designed keeping in mind the study objectives and the high-risk study population. A thorough literature review was performed, and the questions were drafted to cover most of the risk factors. The questions were then translated into the native language and reverse translated. The questionnaire was then pilot tested and validated before administering it to the study population. MSW then conducted interviews with the consenting FSWs using the structured questionnaire. Data on socio demographic characteristics (age, place of birth and marital status), sexual and health seeking behaviour, reproductive health (number of pregnancies, children, termination of pregnancies, type of contraceptives and barriers methods used), tobacco habits and so on was collected from them.

### Screening of FSWs

The screening clinics were conducted at the STD Clinics once in 15 days to recruit participants.

A gynaecologic examination was performed on all participants. Per speculum examinations were conducted to obtain cervical cells for HPV DNA assay by scraping the cervix. Detection of precancer lesions was done by visual inspection with 5% acetic acid (VIA) and cervical smears were obtained from all participating women on the same day they were interviewed.

### HPV DNA hybrid capture II (HC II)

HC II assay kits were procured from the USA. The specimens were collected and stored at −20º until further processing at the HPV laboratory at the tertiary hospital using the HC II microtiter assay in accordance with the manufacturer’s instructions. Cervical samples were classified as positive for DNA from the high-risk HPV types (HPV types 16, 18, 31, 33, 35, 39, 45, 51, 52, 56, 58, 59 and 68) if the relative light unit reading obtained from the luminometer of the HC-II assay equipment was equal or greater than the mean of the positive control values supplied by the HC II kit.

### Visual inspection with 5% acetic acid

The visual screening test VIA was administered by trained health care workers by application of 5% acetic acid to the cervix and visualizing the cervix with the help of a halogen focus lamp. VIA was considered to be positive if definite acetowhite lesions were visualized close to the squamocolumnar junction.

### Colposcopy with or without biopsy

Colposcopy with or without biopsy was administered to all the participants irrespective of the status of VIA screening test. After the collection of cervical samples for HPV testing and VIA, colposcopy was performed by trained doctors and the colposcopy impression was noted down along with a punch biopsy from the acetowhite area on the cervix.

The true disease was defined as a histologically confirmed high-grade Squamous Intraepithelial Lesion. The consensus category includes cervical intraepithelial lesion (CIN) 1, 2 and 3 and/or carcinoma *in situ*.

### Post-test counselling

At the end of all testing procedures, posttest counseling was done by the doctor and MSW to explain the significance and the results of the testing procedures performed. In addition, tobacco users were offered tobacco cessation counseling by the MSW. The importance of follow-up visits to understand the results of HPV or cervical biopsy were explained to the women.

### Data management and analysis

Data were entered and analyzed using Statistical Package for the Social Sciences v 29. Data were regularly checked for consistency, safety and analysis at regular intervals. Frequencies of sociodemographic, reproductive and sexual behaviour attributes were determined. Prevalence of HPV infection, disease spectrum of CIN and risk factors for acquiring HPV infection with 95% CI were estimated.

## Results

A total ten NGOs were identified out of which six consented for participation in the project. Around 12 sensitization programs and 39 cervical cancer awareness programs were held at community settings. A total of 736 eligible FSWs were contacted, counselled and were invited for cervical cancer awareness and screening. A total of 448 (60.8%) FSWs consented to the program.

### Sociodemographic characteristics

Table 1 shows the socio-demographic details of the study population. More than half of the of the FSWs were in the age bracket of 18–30 years (58.0%), single (58.7%) with no formal education (66.5%) and practiced Hinduism (58.3%). Tobacco consumption habit was found in most of the FSWs (51.8%), of which 95.7% were smokeless tobacco consumers. The majority (87.5%) had worked as sex workers and the rest (12.5%) worked in bars.

### Sexual behaviour profiling

Table 2 shows sexual behaviour profiling of the study population. Around half of the participants were aged less than 18 years when they had their first sexual exposure (49.8%). A sizeable percentage had started work as commercial sex workers at less than 18 years of age (21.7%). The mean number of clients seen per day was more than four for 23.8% of the participants. Almost half (50.4%) of these workers have been working as FSWs for more than 4 years.

Barrier contraception was consistently used by 60.0% of the FSWs for all sexual acts. Among those who used barrier contraceptives (BCs) inconsistently, the most common reason for non-use was the lack of awareness of the risks associated with not using barrier contraception (63.3%) followed by an objection by the partner (34.0%) for use.

Regarding awareness of STDs, the majority (76.6%) had attended an awareness program for the prevention of these diseases. However, among those who had themselves suffered from symptoms of STI (57.4%), most did not seek any treatment (58%) nor did they ever get themselves tested for HIV-acquired immunodeficiency syndrome (AIDS) at a healthcare facility (64.3%).

### Results of screening, histopathology tests

Table 3 shows the results of screening and histopathology tests performed for cervical cancer. The prevalence of HPV DNA positivity was 35.5%. The VIA (visual inspection by acetic acid) was positive in 12.5% FSWs. Histopathology examination of the cervix revealed CIN I in four participants, CIN II in five participants and CIN III in one participant. The overall prevalence of CIN was 2.2%.

### Predictors of prevalence of HPV infection

Table 4 shows the predictors of positive HPV infection among the participants. Factors significantly associated with HPV DNA positivity were age group younger than 30 years (odds ratio (OR) = 2.098, 95% CI 1.408–3.127), Illiteracy (OR = 2.015, 95% CI 1.305–3.112), being single (OR = 2.409, 95% CI 1.558–3.724), less than 18 years of age at the time of initiating work as FSW (OR = 3.718, 95% CI 3.718–2.392), having more than five clients per day (OR = 2.078, 95% CI 1.301–3.318), been working as an FSW for more than 5 years (OR = 2.321, 95% CI 1.455-3.701), not using barrier contraception methods (OR = 5.155, 95% CI 3.395–7.827) and having no exposure to HIV/AIDS education program (OR = 29.153, 95% CI 15.385–55.240).

### Risk of CIN among HPV-positive FSWs

Table 5 shows the risk of CIN among those who are HPV positive. As observed from the study results among those who were HPV positive, histopathology examination of the cervix found CINI in three participants, CIN II in four participants and CIN III in one participant. A significant association between a positive HPV DNA test and the presence of CIN is also observed. Individuals with a positive HPV DNA test are substantially more likely to have CIN compared to those with a negative test. The OR with 95% C.I. of 7.6 (1.59–36.25), with a statistically significant *p*-value of 0.0109 shows the effectiveness of the HPV DNA test as a screening tool for CIN.

## Discussion

This study found the overall prevalence of HPV DNA positivity rate among sample as 35.5%. This is higher than the prevalence found in studies done in other parts of India. A study by Sarkar *et al* [22] in West Bengal found an HPV prevalence of 25% and Singh *et al* [23] in Chandigarh reported HPV prevalence of 27.5%. Similarly, studies conducted in African countries such as Ghana (26%) and Togo (32.9%) have also reported a lower prevalence [24, 25]. However, higher HPV prevalence was noted in the studies done in other countries: Vietnam (85.0%), Cambodia (41.1%), Belgium (41.7%) and Dominican Republic (43.4%) [26–29]. The systematic review by Soohoo *et al* [30] and Wu *et al* [31] show a higher prevalence of HPV at 42.7% and 39.5%, respectively. Detailed discussion on major predictors of the prevalence of HPV among respondents of this study are as follows:

In this study, more than half (58.0%) of the females were in the age group of 18–30 years. It is observed that those who are HPV positive were two times more likely to be less than 30 years of age (OR = 2.098, 95% CI 1.408–3.127). These results are consistent with those reported in a number of other studies [22, 28, 30]. This is also in accordance with the literature which shows that the probability of HPV decreases significantly with growing age [32]. The study conducted in Vietnam and Mexico, however, did not show any change in HPV prevalence by age [26, 33].

This study found that 66.5% of the FSWs with no formal education. Similar proportions of illiteracy were reported in the study by Sarkar *et al* [22] in West Bengal (62.9%), Singh *et al* [23] in Chandigarh (60.8%) and Hernandez and Nguyen [26] in Vietnam (63%). Our study shows that HPV positivity is two times more common among Illiterates (OR = 2.015, 95% CI 1.305–3.112), similar to the study of Hernandez and Nguyen [26]. Lack of education among women makes them less receptive to health awareness programs and prevents them from taking informed decisions about their sexual and reproductive health, thereby predisposing them to HPV infection. We also observed that 40.0% of FSWs did not use BCs consistently for all commercial sexual acts. This percentage is lesser than that reported in studies by Hernandez and Nguyen [26] (84%), Couture *et al* [27] (90.8%) and Singh *et al* [23] (71.6%). Our findings also show that participants with HPV DNA positivity were unlikely to be using barrier contraception methods (OR = 5.155, 95% CI 3.395–7.827) and have had any exposure to HIV/AIDS education program (OR = 29.153, 95% CI 15.385–55.240).

Literature shows that tobacco causes diminished antibody reaction in HPV16/18-afflicted young females [34]. Tobacco use in our study is reported as 51.8%, of which 95.7% were smokeless tobacco consumers. However, no association was found between HPV status and tobacco use in this study. Similar results have been reported by Jia *et al* [35]. However, a study by Singh *et al* [23] shows higher HPV positivity among FSWs who smoked tobacco (36.7% versus 24.4%, OR = 4.11, *p* = 0.05).

It is observed in the study that younger age at first sexual exposure was not significantly associated with HPV positivity, but it was associated with participants who started as FSW at an age younger than 18 years. This could be because age at first sexual contact is more likely to be a single sexual partner exposure. However, it is likely that when participants started working as FSWs the exposure to multiple sexual partners because of commercial sex work resulted in increased HPV positivity in this group. Sarkar *et al* [22] showed found that sex workers beginning their work at ≤20 years of age had the highest HPV prevalence (29.7%), followed by older age groups. Females with early initiation of sexual intercourse may get infected with the HPV virus earlier in their life course thus giving the virus more time to persist and progress to initiate precancer changes.

We found a positive association between more than five clients per day and HPV-positive status (OR = 2.078, 95% CI 1.301–3.318). Likewise, Sarkar *et al* [22] observed that sex workers having the usual number of four or more clients per day were nearly four times more likely of getting HPV (OR = 3.9; 95% CI 1.6–9.4). Couture *et al* [27] noted a higher number of sexual partners (AOR 1.05; 95% CI: 1.01–1.09) to be associated with HPV infection. Contrary to this, Hernandez and Nguyen [26] in their study noted that HPV infection was lower among FSWs attending to more clients per day. They suggested that constant, recurrent contact with HPV enhances the immunogenic reaction locally. Hence, FSWs with the most clients are relatively more protected from getting infected by the new HPV.

Taking into consideration the length of time as a sex worker, our study found that FSWs working for more than 5 years had a significantly greater risk of acquiring HPV infection (OR = 2.321, 95% CI 1.455–3.701). This could be due to acquired immunity gained against HPV antigen over time. This is contrary to the findings by Sarkar *et al* [22] which show sex workers with a lesser duration of work are at an increased chance of HPV acquisition (OR = 3.3, 95% CI 1.455–7.6).

Histopathology examination of the cervix revealed CIN I in 0.9%, CIN II in 1.1% and CIN III in 0.2% participants, overall prevalence being 2.2%. The findings are higher compared to the study by Sarkar *et al* [22] where 1% of the studied FSW population suffered from a pre-cancerous lesion caused by high-risk HPV. However, the CIN prevalence in our study was lower compared to the study done in Pune, Ahmednagar and Sangli districts of Maharashtra, India (8.3%) by Joshi *et al* [36]. This may be because these districts have a high prevalence of HIV infection as well. The CIN prevalence in our study was lower when compared to those reported from Kenya (5.5%), Cameroon (5.9%) and China (16.82%) [36, 27, 38]. Our study found that those who were HPV Positive had seven times (OR = 7.60, 95% CI 1.59–36.25) increased risk of getting CIN. The review published by Muñoz *et al* [39] which analysed data from 11 case control studies across 9 countries showed that while the odds of developing cervical cancer vary with the type of HPV infection, the overall risk of cervical cancer is much higher (OR = 158.2, 95% CI, 113.4 to 220.6) with oncogenic type [39]. Our study shows a high prevalence of HPV among FSWs and its strong association with CIN emphasizing the need for enhanced preventive measures and cervical cancer screening for this high-risk population.

## Strengths and limitations

Our study demonstrates several strengths of the program. The study was conducted aligning with the other health program and welfare measures targeted towards the FSW community by the local non-government organisations. Peer educators from the FSW community were identified and sensitised for encouraging and counselling the FSW to participate in the cervical cancer screening program. The study thus parallelly demonstrates the feasibility of introducing cervical cancer screening along with interventions for HIV/AIDS and STD control and prevention during gynaecological examinations targeted for STD intervention. We report the following limitations of our study. Since HPV detection was undertaken by a qualitative test (HC II) therefore the high-risk HPV genotype could not be ascertained among this at-risk population. Also, the HIV status of this high-risk population could not be captured due to reasons of confidentiality and hence the association between the HIV status and HPV infection and vice versa could not be established. Due to the cross sectional nature of the study, the risk of progression to CIN and cervical cancer could not be ascertained.

## Conclusion

FSWs have a high prevalence of HPV infection and are at increased risk of cervical cancer. Cervical cancer awareness and screening is not part of any health care interventions currently targeted towards this high-risk group. Because of their similar epidemiological determinants, the scope of the current national program for STD/HIV AIDS prevention and control should be expanded to cover cervix cancer prevention and screening which will be highly cost-effective to decrease the burden of cervical cancer among FSW.

## List of abbreviations

BC, Barrier contraceptive; CI, Confidence interval; CIN Cervical intraepithelial neoplasia; FSWs, Female sex workers; HC II, Hybrid capture II; HIV/AIDS, Human immunodeficiency virus/acquired immunodeficiency syndrome; HPV, Human papilloma virus; IRB, Institutional Review Board; MSW, Medical social worker; NGO, Non-governmental organization; OR, Odds ratio; SIL, Squamous intraepithelial lesions; STD, Sexually transmitted diseases; VIA, Visual inspection by acetic acid.

## Conflicts of interest

The authors declare no conflicts of interest in this work.

## Author contributions

SP designed and implemented the study, interpreted the data and prepared the manuscript; VP prepared the manuscript; GM contributed in data analysis and interpretation; SKB and KD were involved with HPV and histology reporting, respectively, and interpretation of data and preparation of the manuscript.

## Figures and Tables

**Table 1.** Sociodemographic profiling of FSWs (*N* = 448).

Sociodemographic characteristics		Frequency (percentage)
Age (in years)	18–30	238 (58.0)
	31–40	144 (32.1)
	>40	66 (14.7)
Education	Nil	298 (66.5)
	Primary School	29 (6.5)
	Middle School	71 (15.8)
	High School and above	40 (11.2)
Religion	Hindu	261 (58.3)
	Muslim	177 (39.5)
	Christian	4 (0.9)
	Other	6 (1.3)
Occupation	Bar	56 (12.5)
	Sex worker	392 (87.5)
Age of Menarche (in years)	≤10	5 (1.1)
	11–13	367 (81.9)
	14–15	59 (13.2)
	>15	17 (3.8)
Marital status	Married and living with husband	132 (29.5)
	Married but not living with husband	26 (5.8)
	Living with sexual partner	27(6.0)
	Currently single	263 (58.7)
Age at marriage (*n* = 158) (in years)	<18	97 (61.3)
	≥18	61 (38.6)
Pregnancies	0	51 (11.4)
	1–2	183 (40.8)
	>2	214 (47.8)
Abortions	0	268 (59.8)
	1–2	145 (32.4)
	>2	35 (7.8)
Tobacco uses in any form	Yes	232 (51.8)
	No	216 (48.2)
If yes, type of tobacco consumption (*n* = 232)	Smokeless	221 (95.7)
	Smoking	9 (3.5)
	Smoking/smokeless	2 (0.9)

**Table 2.** Sexual behaviour profiling of FSWs (*N* = 448).

Sexual behaviour characteristics		Frequency (percentage)
Age of first sexual exposure (in years)	<18	223 (49.8)
	18–21	138 (30.8)
	>21	17 (3.8)
	Refused to give information	70 (15.6)
Mean number of clients per day	<5	231 (51.5)
	≥5	107 (23.8)
	Refused to give information	110 (24.6)
Age at which started commercial sex work (in years)	<18	97 (21.7)
	18–21	142 (31.9)
	>21	137 (30.6)
	Refused to give information	71 (15.8)
Duration worked as a commercial sex worker (in years)	<5	132 (29.5)
	≥5	226 (50.4)
	Refused to give information	90 (20.1)
Usage of BC	Consistent (for all sexual acts)	269 (60.0)
	Inconsistent or no use	179 (40.0)
Reasons for non-usage of BC among inconsistent and non-users (*n* = 179)	Partner objection	61 (34.0%)
	Non availability	5 (2.7%)
	Unaware about risk perception	113 (63.3%)
Does FSW insist on condom use?	Yes	310 (69.2)
	No	130 (29.0)
	Sometimes	8 (1.8)
Exposure to STD/HIV/AIDS awareness program in the past	Yes	343 (76.6)
	No	105 (23.4)
Awareness about method of prevention of STD/HIV/AIDS	Condom use	317 (70.7)
	Single faithful and uninfected partner	2 (0.4)
	Both	10 (2.2)
	Not aware	119 (26.5)
Perceives risk of HIV/AIDS infection	Yes	320 (71.4)
	No	128 (28.6)
Symptoms of STI in past 12 months	Genital discharge	228 (50.9)
	Genital ulcer	7 (1.6)
	Burning pain during micturition	22 (4.9)
	Nil/none	191 (42.6)
Sought health facility for diagnosis or treatment of STD	Yes	108 (42.0)
	No	149 (58.0)
Self-testing for HIV/AIDS at health care facility	Yes	160 (35.7)
	No	288 (64.3)

BC, Barrier contraceptive; STD, Sexually transmitted disease; HIV, Human immunodefeciency virus; AIDS, Acquired immunodeficinecy syndrome; STI, Sexually transmitted infection

**Table 3.** Results of screening and histopathology tests done among FSWs (*N* = 448).

Screening and histopathology tests		n (%)
HPV DNApositive		159 (35.5)
VIApositive		56 (12.5)
Histopathology	Inflammation atypia	37 (8.3)
	CIN I	4 (0.9)
	CIN II	5 (1.1)
	CIN III	1 (0.2)

HPV, Human papilloma virus; VIA, Visual inspection by acetic acid; CIN, Cervical intraepithelial neoplasia

**Table 4.** Predictors of prevalence of HPV infection among FSWs: unadjusted bivariate logistic regression analysis (*N* = 448).

Characteristics	Total	HPV positive *n* (%)	OR	95% CI	*p* value
Age group (in years )					
> 30	210	56 (26.7)	1		
≤30	238	103 (43.3)	2.098	1.408–3.127	<0.001
Education					
Educated	150	38 (25.3)	1		
Illiterate	298	121 (40.6)	2.015	1.305–3.112	0.002
Occupation					
Bar worker	56	16 (28.6)	1		
Sex worker	392	143 (36.5)	1.436	0.776–2.656	0.249
Marital status					
With spouse	159	37 (23.3)	1		
Single	289	122 (42.2)	2.409	1.558–3.724	<0.001
Age at marriage (in years )					
≥18	61	14 (23.0)	1		
<18	97	22 (22.7)	0.985	0.459–2.112	0.969
Tobacco use					
No	216	84 (38.9)	1		
Yes	232	75 (32.3)	0.751	0.509–1.106	0.147
Pregnancies					
≤2	234	105 (44.9)	1		
>2	214	54 (25.2)	0.415	0.277–0.620	<0.001
Abortions					
≤2 No Abortions	268	107 (39.9)	1		
>2 Abortions	180	52 (28.9)	0.611	0.408–0.916	0.017
Age at first sexual exposure (in years )					
≥18	155	62 (40.0)	1		
<18	223	77 (34.5)	0.791	0.518–1.208	0.278
Age at starting as FSW (in years )					
≥18	225	55 (24.4)	1		
<18	152	83 (54.6)	3.718	3.718–2.392	<0.001
No. of clients/day					
<5	231	76 (32.9)	1		
≥5	107	54 (50.5)	2.078	1.301–3.318	0.002
Duration in years FSW (in years )					
<5	132	35 (26.5)	1		
≥5	226	103 (45.6)	2.321	1.455–3.701	<0.001
Use of BC					
Yes	269	56 (20.8)	1		
No use	179	103 (57.5)	5.155	3.395–7.827	<0.001
Exposure to HIV/AIDS education					
Education	343	67 (19.5)	1		
No Education	105	92 (87.6)	29.153	15.385–55.240	<0.001
STI symptoms					
No symptoms	257	77 (30.0)	1		
Symptoms	191	82 (42.9)	0.569	0.384–0.841	0.005
Sought treatment for STI					
Treatment	191	58 (30.4)	1		
No treatment	257	101 (39.3)	1.485	0.998–2.209	0.051
Self-initiated testing for HIV					
Yes	160	49(30.6)	1		
No	288	110 (38.2)	1.400	0.927–2.113	0.109

*p* value of ≤0.05 was considered to be statistically significant

FSW, Female sex workers; BC, Barrier contraception; HIV, Human Immunodeficiency Virus; AIDS, Acquired Immunodeficiency Virus; OR, Odds ratio; CI, Confidence interval

**Table 5.** Risk of CIN among HPV positive FSWs.

Screening tests	CIN present n(%)	CIN absent n(%)	OR	95% CI	p value
**HPV DNA**					
Positive	8 (80.0)	151 (34.5)	7.60	1.59–36.25	0.0109*
Negative	2 (20.0)	287 (65.5)	1		

*p* value of ≤0.05 was considered to be statistically significant

OR, Odds ratio; CI, Confidence interval; CIN, Cervical intraepithelial neoplasia
